# Supplementary material for: Regulation of PDGF-BB signaling in placental pericytes by soluble PDGFRβ isoforms: implications for fetoplacental vascular development
Source: Angiogenesis. 2026 Sep 3;29(4):67. doi: 10.1007/s10456-026-10091-3 (PMC13541826; doi:10.1007/s10456-026-10091-3)
Supplement: Supplementary file 1 [file 10456_2026_10091_MOESM1_ESM.pdf]

Supplemental Figure 1. Plots of gene expression values represented as average counts per million (CPM) like values for the genes indicated, from Arutyunyan et al. Nature 2023. Blue arrowheads indicate a perivascular cell population that likely contains human placental pericytes.

Supplemental Figure 2. Murine Chorio-allantoic Vasculature Progressively Expands during Gestation, with the Ratio of Pericytes to Endothelial Cells Remaining Relatively Constant. (A) Graph of PECAM-1-positive cells normalized to all DAPI-positive cells for a given image from the indicated time points, E11.5 (n=3, green bar), E14.5 (n=3, orange bar), and E18.5 (n=4, purple bar). Individual data points shown, and errors bars are standard deviation. (B) Graph of the ratio between NG2-positive cells and PECAM-1-positive cells for a given image from the indicated time points, E11.5 (n=3, green bar), E14.5 (n=3, orange bar), and E18.5 (n=4, purple bar). Individual data points shown, and errors bars are standard deviation.

Supplemental Figure 3. Target Gene Expression Normalized to a Vessel-Based Gene or a Cell Type-Specific Gene for Relative Comparison. In (A-E), PC marker gene expression shown in Figure 2B-F was normalized to *Kdr/Flk1* to demonstrate how PC gene expression varies relative to the level of vascular growth across gestation. *Note in (A-E), for each gene expression graph shown: all data were normalized to Kdr/Flk1, Tbp served as the housekeeping gene, individual data points were plotted for each embryonic day with averages represented by bars: E9.5--green, E14.5--orange, E18.5--purple, error bars represent standard error of the mean, and *p≤0.05 by one-way ANOVA with post-hoc Tukey test for comparisons indicated.* (C) Graph of relative *Cspg4* expression within the murine placenta at E9.5 (n=5), E14.5 (n=6), and E18.5 (n=6). (D) Graph of relative *Acta2 (αSMA)* expression within murine placenta at E9.5 (n=6), E14.5 (n=6), and E18.5 (n=6). (E) Graph of relative *Full Length (FL)-Pdgfrb* expression within murine placenta at E9.5 (n=6), E14.5 (n=6), and E18.5 (n=6). (F) Graph of relative *Vitronectin (Vtn)* expression within murine placenta at E9.5 (n=6), E14.5 (n=6), and E18.5 (n=5). (G) Graph of relative *Notch3* expression within murine placenta at E9.5 (n=6), E14.5 (n=5), and E18.5 (n=5). In (F-J), gene expression shown in Figure 3A-F was normalized to a cell-specific gene to demonstrate expression changes over time relative to that population. *For each gene expression graph shown: Tbp served as the housekeeping gene, individual data points were plotted for each embryonic day with averages represented by bars: E9.5--green, E14.5--orange, E18.5--purple, error bars represent standard error of the mean, and *p≤0.05 by one-way ANOVA with post-hoc Tukey test for comparisons indicated. In (F-G), expression was normalized to Kdr/Flk1:* (F) Graph of relative *Flt-1* expression within murine placenta at E9.5 (n=6), E14.5 (n=6), and E18.5 (n=5). (G) Graph of relative *Pdgfb* expression within murine placenta at E9.5 (n=6), E14.5 (n=6), and E18.5 (n=6). *In (H), expression was normalized to Notch3:* (H) Graph of relative *soluble Pdgfrβ* expression (intron4 truncation variant) within murine placenta at E9.5 (n=6), E14.5 (n=5), and E18.5 (n=6). *In (I-J), expression was normalized to Gata3:* (I) Graph of relative *Vegfa* expression within the murine placenta at E9.5 (n=4), E14.5 (n=6), and E18.5 (n=6). (J) Graph of relative *PlGF* expression within murine placenta at E9.5 (n=6), E14.5 (n=5), and E18.5 (n=6).

Supplemental Figure 4. Ratios of sPDGFRβ to full-length PDGFRβ were Calculated for All Relevant Data Sets (Protein and mRNA Transcripts) in Figure 2-6. (A-B) Graphs of sPDGFRβ to full-length PDGFRb for murine placenta gene expression (A) and protein abundance (B). *For gene expression graph shown in (A), Tbp served as the housekeeping gene. In both (A) and (B), individual data points were plotted for each embryonic day with averages represented by bars: E9.5--green, E14.5--orange, E18.5--purple, error bars represent standard error of the mean, and *p≤0.05 by one-way ANOVA with post-hoc Tukey test for*

comparisons indicated. (C) Heatmap of the ratios of integrated densities for bands at ~55 kDa (presumed to be sPDGFRβ) relative to bands at ~160 kDa (interpreted as full-length PDGFRβ) and normalized to the average ratio of all replicates for a given subject. The reference bar depicts the color-based gradient representing lower ratios (red) to higher ratios (green), with moderate ratios in the yellow range. (D-E) Graphs of sPDGFRβ to full-length PDGFRβ for murine embryonic stem cell protein abundance (D) and gene expression (E). *For gene expression graph shown in (E), Tbp served as the housekeeping gene. In both (D) and (E), individual data points were plotted for each differentiation day with averages represented by bars: dd8--tan, dd10--green, dd12--blue, error bars represent standard error of the mean, and *$p \le 0.05$ by one-way ANOVA with post-hoc Tukey test for comparisons indicated.* (F-H) Graphs of sPDGFRβ to full-length PDGFRβ for murine embryonic stem cell protein abundance. *In all graphs, individual data points were plotted for each treatment group with averages represented by bars, and error bars represent standard error of the mean: (F) DMSO Tx--purple, ADAM10 Tx—pink; (G) control—blue, n=3 for each time point, 3% O2 hypoxia—yellow, n=3 for each time point; (H) EGM Basal Form control—green, n=3; EGM Basal Form + VEGF-A group—orange, n=3; EGM Basal Form + PDGF-BB group—brown, n=3; EGM Basal Form + Complete EGM Kit group—gray, n=3.*

Supplemental Figure 5. 3D Surface Plots in Support of Data Presented in Figure 6E. Three-dimensional (3D) surface plots of the relative signal intensity of the phospho-PDGFRb bands within the areas outlined by yellow dotted rectangles in (A). To optimize visualization of signal intensity maximums and minimums, regions within the blots were rotated ~112° (B). Arrowheads indicate the corner in the blot that corresponds to the same corner in the 3D signal intensity surface plot following rotation. These plots were generated in ImageJ/FIJI for the blot images presented in Figure 6E using the “3D Surface Plot” tool within the “Analyze” menu.

Supplemental Figure 6. Original western blots in support of Figure 3G.

Supplemental Figure 7. Original western blots in support of Figure 3I, Baby 001 and 002.

Supplemental Figure 8. Original western blots in support of Figure 3I, Baby 003 and 004.

Supplemental Figure 9. Original western blots in support of Figure 4E (Differentiation Day 8 and half of Differentiation Day 10 samples).

Supplemental Figure 10. Original western blots in support of Figure 4E (Half of Differentiation Day 10 and Differentiation Day 12 samples).

Supplemental Figure 11. Original western blots in support of Figure 5B.

Supplemental Figure 12. Original western blots in support of Figure 5B.

Supplemental Figure 13. Original western blots in support of Figure 5E.

Supplemental Figure 14. Original western blots in support of Figure 5E.

Supplemental Figure 15. Original western blots in support of Figure 6B.

Supplemental Figure 16. Original western blots in support of Figure 6B.

Supplemental Figure 17. Original western blots in support of Figure 6E.

Supplemental Figure 18. Original western blots in support of Figure 6E.

Supplemental Figure 19. Original western blots in support of Figure 6E.

Supplemental Figure 1.

A Plots of gene expression values represented as average counts per million (CPM) like values for the genes indicated, from Arutyunyan et al. *Nature* 2023. Blue arrowheads indicate a perivascular cell population that likely contains human placental pericytes.

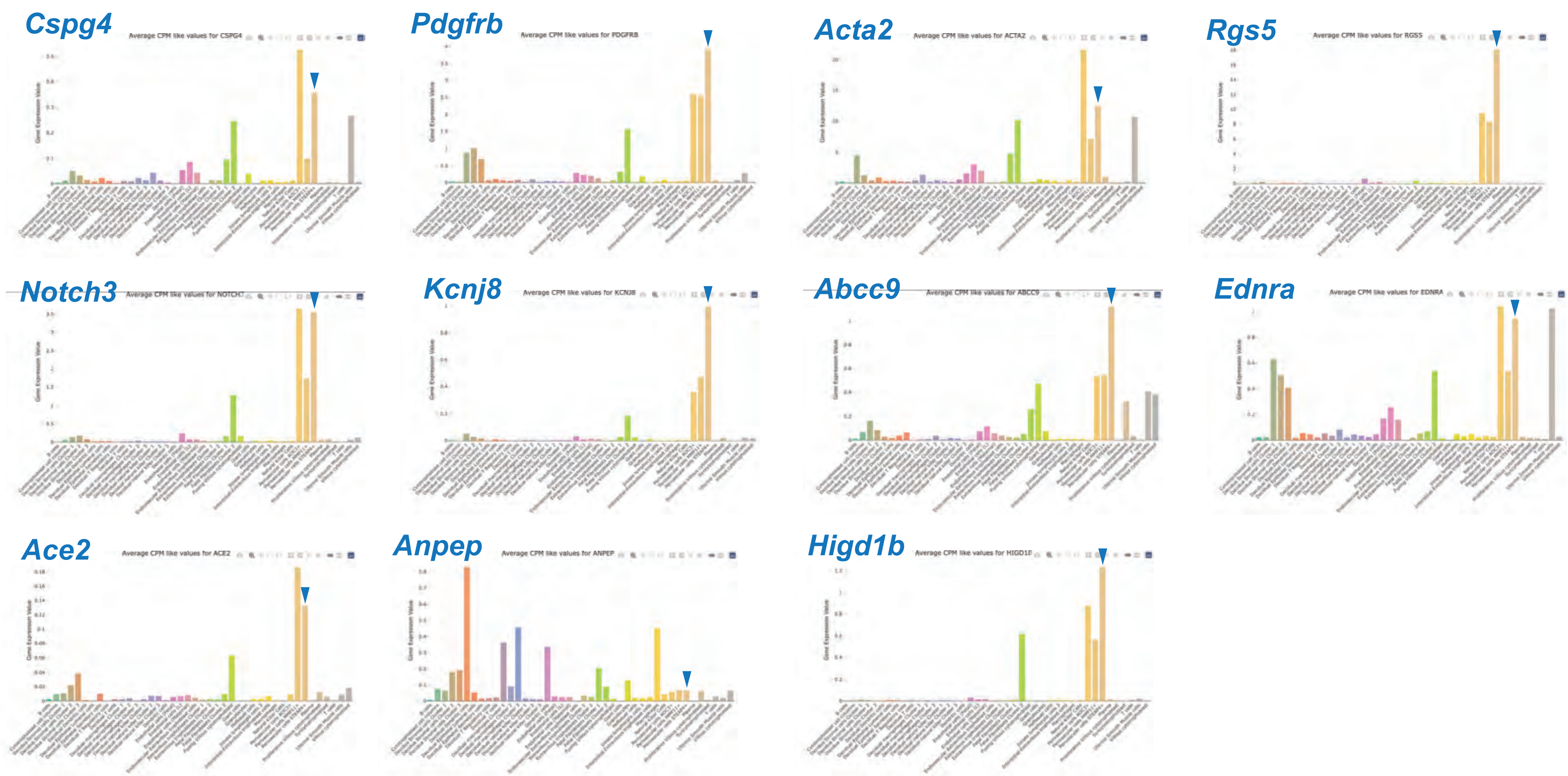

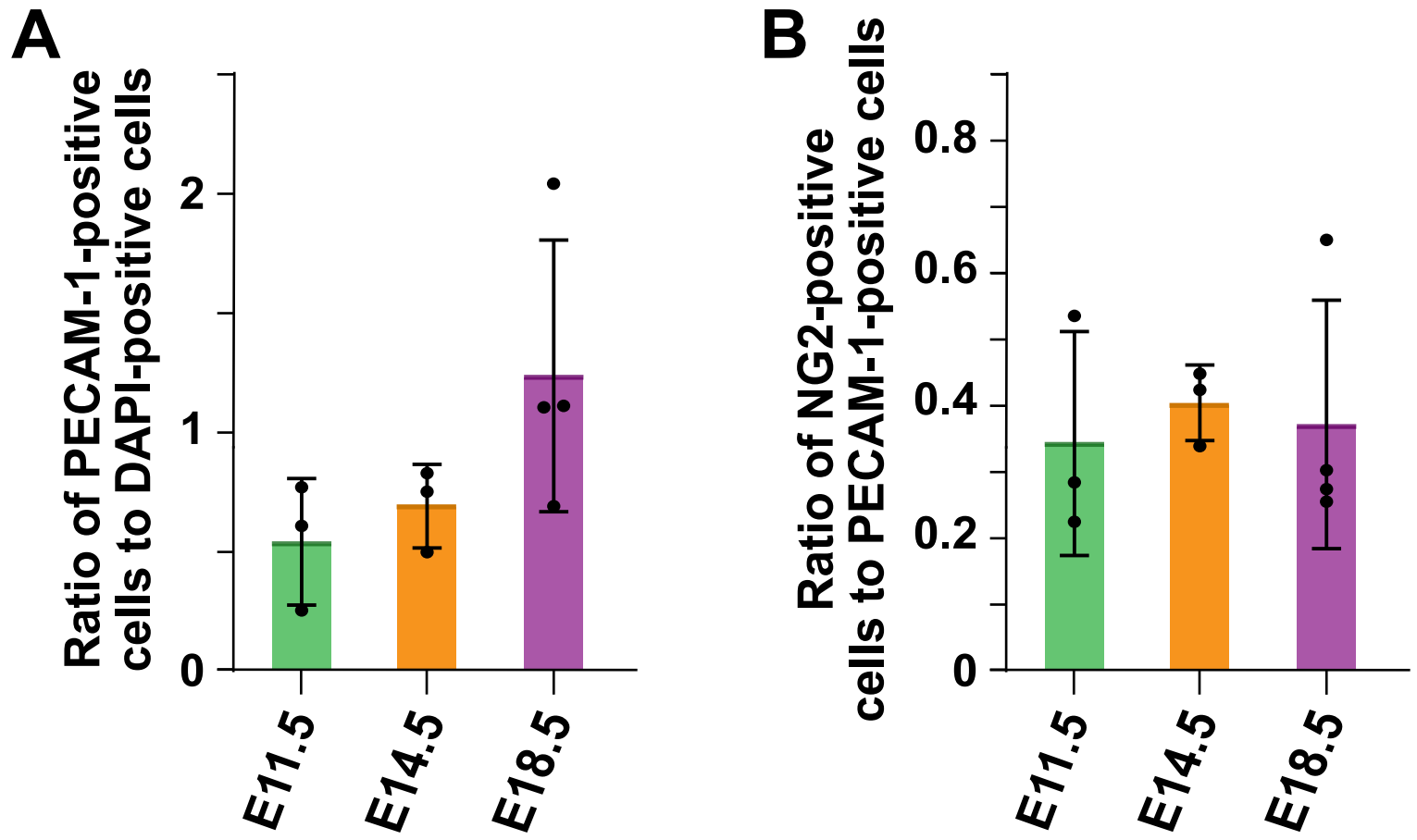

Supplemental Figure 2.

Supplemental Figure 3.

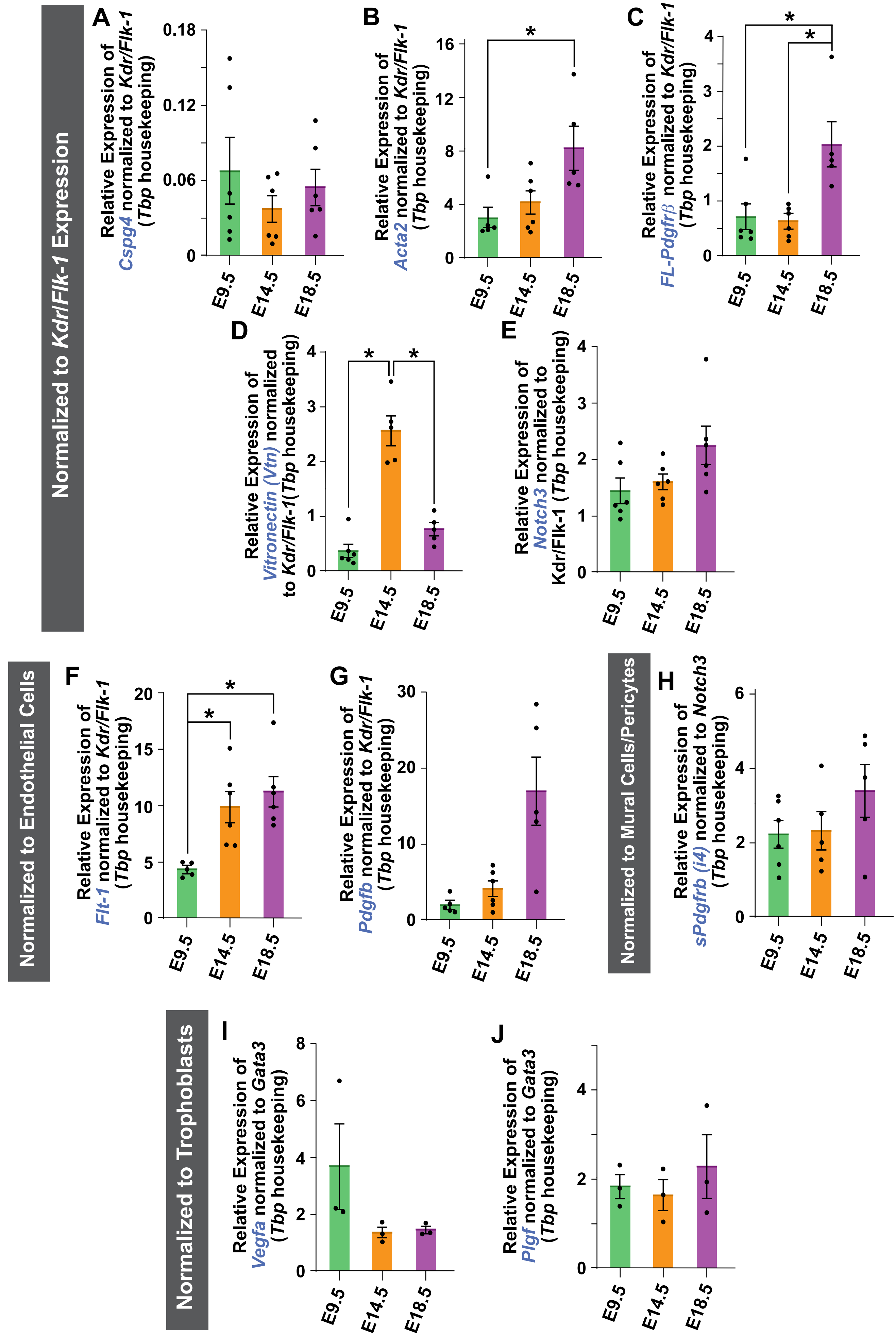

Supplemental Figure 4.

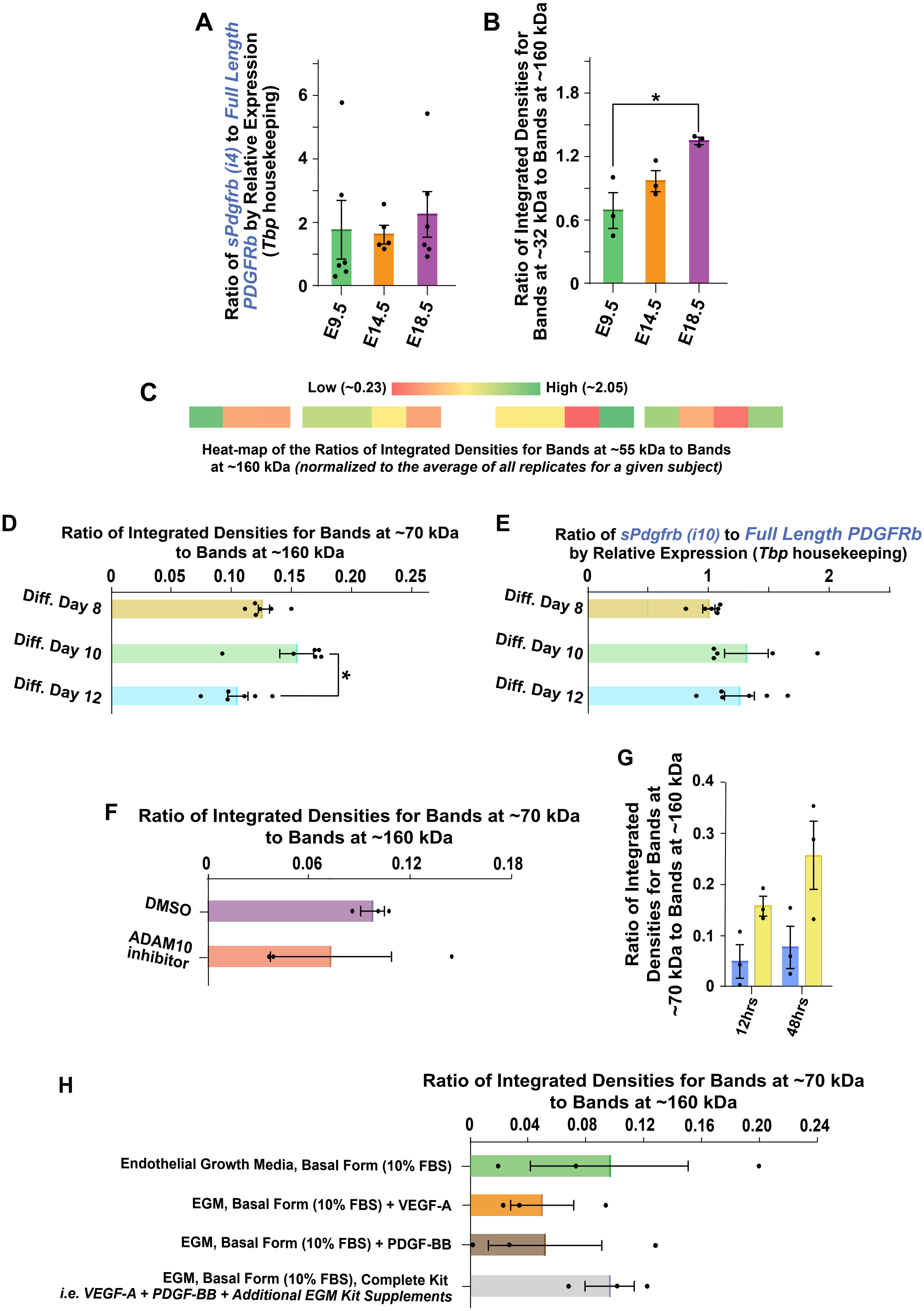

Supplemental Figure 5.

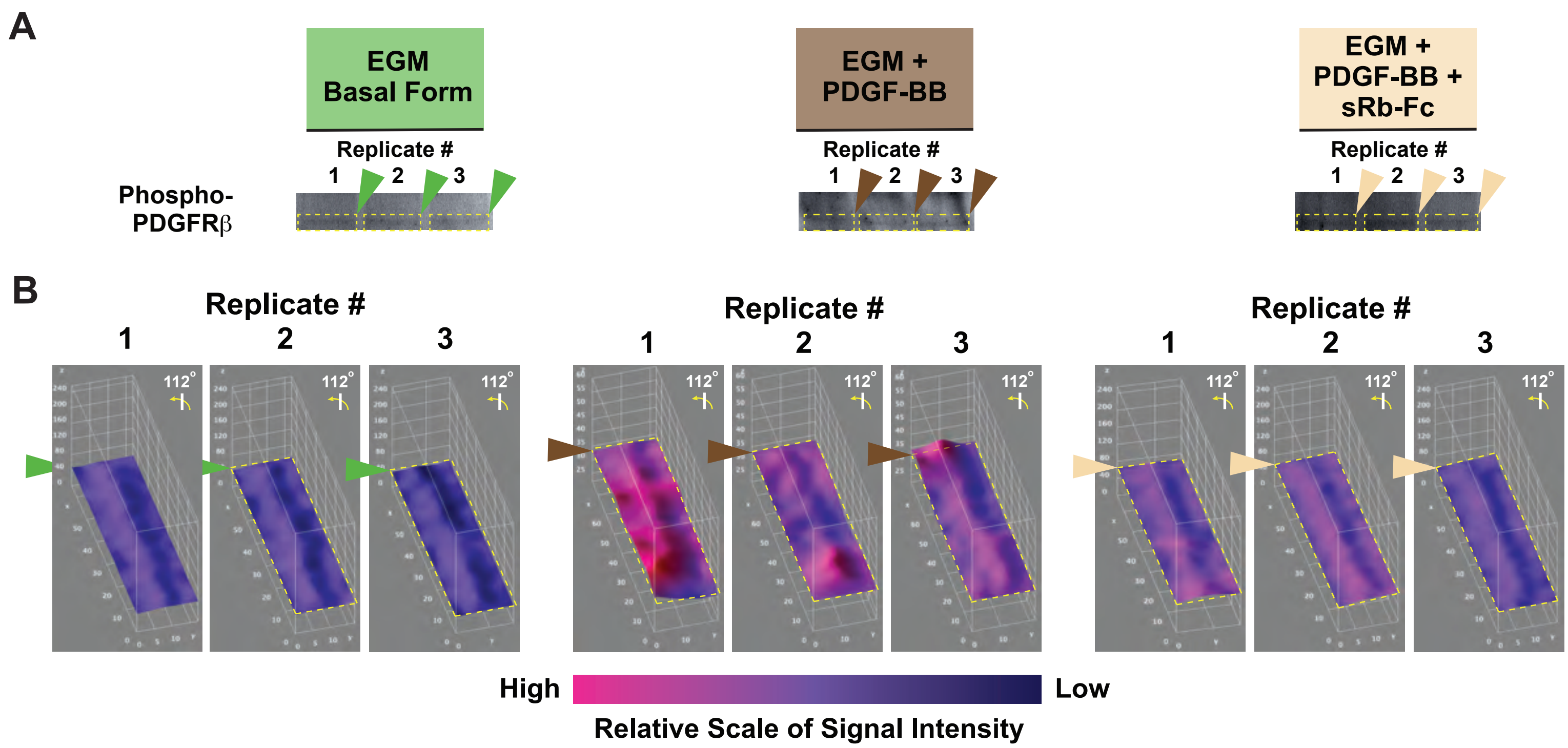

Three-dimensional (3D) surface plots of the relative signal intensity of the phospho-PDGFRb bands within the areas outlined by yellow dotted rectangles in (A). To optimize visualization of signal intensity maximums and minimums, regions within the blots were rotated ~112° (B). Arrowheads indicate the corner in the blot that corresponds to the same corner in the 3D signal intensity surface plot following rotation. These plots were generatedin ImageJ/FIJI for the blot images presented in Figure 6E using the “3D Surface Plot” tool within the “Analyze” menu.

A

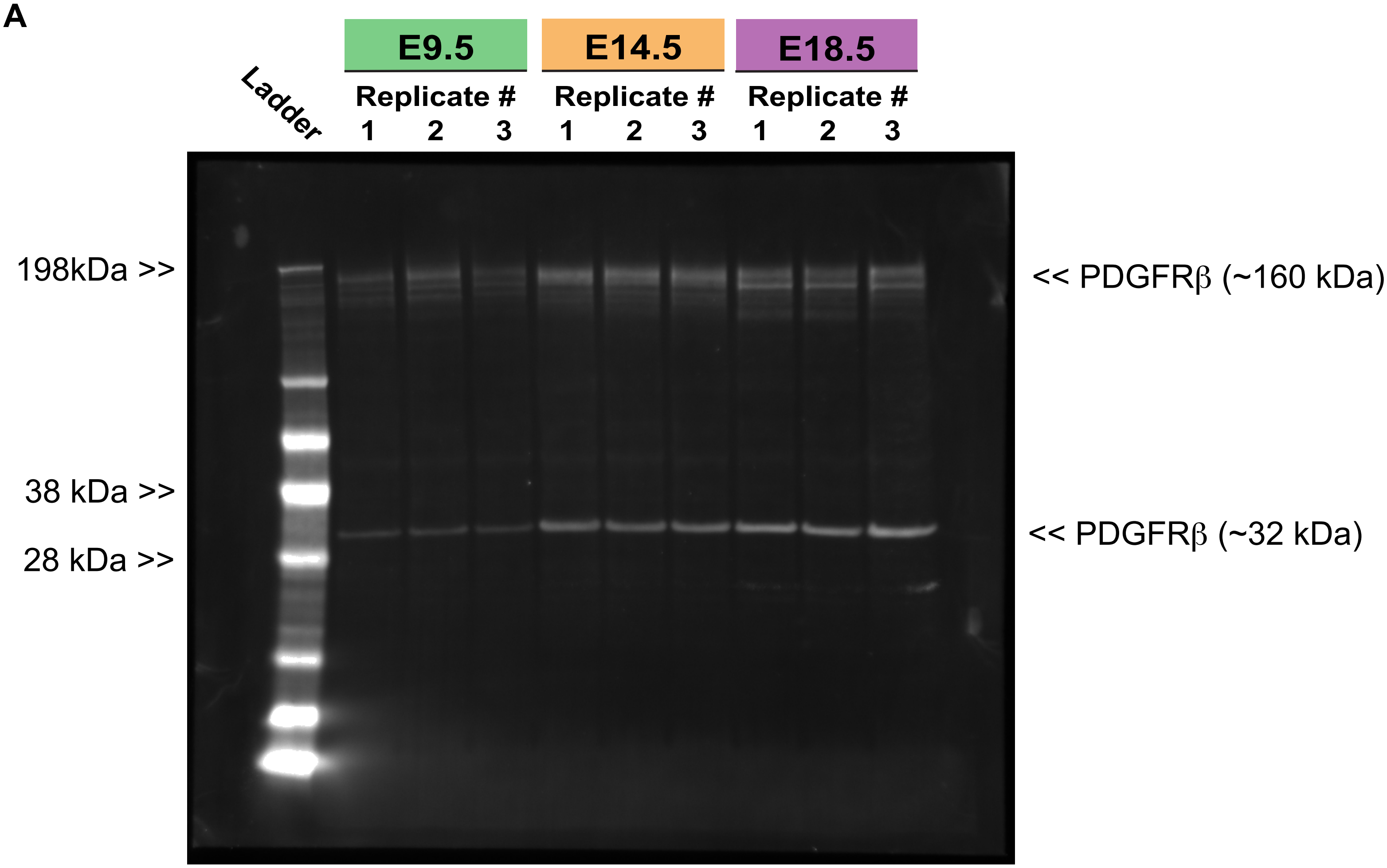

B

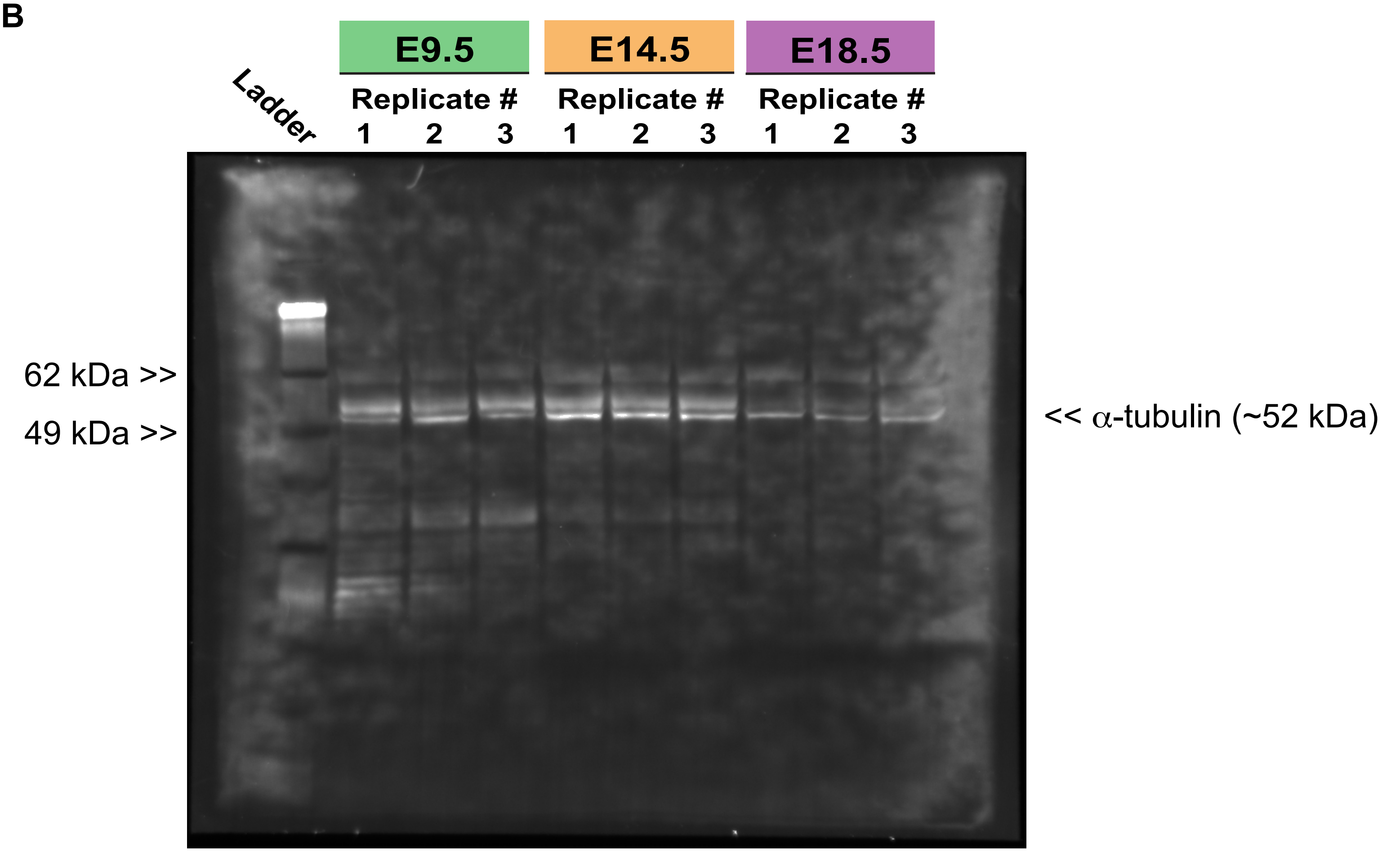

Supplemental Figure 6.

A

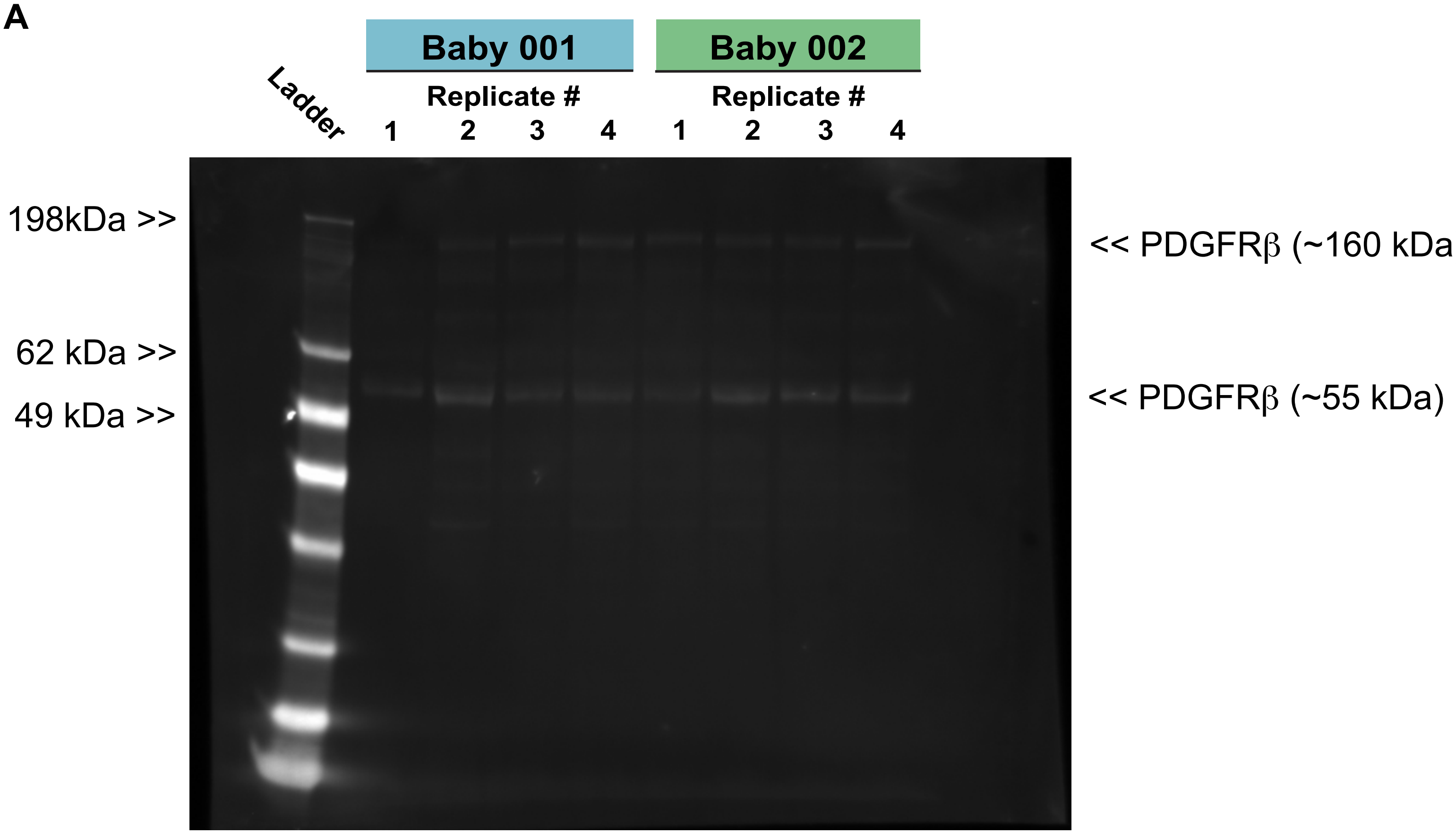

B

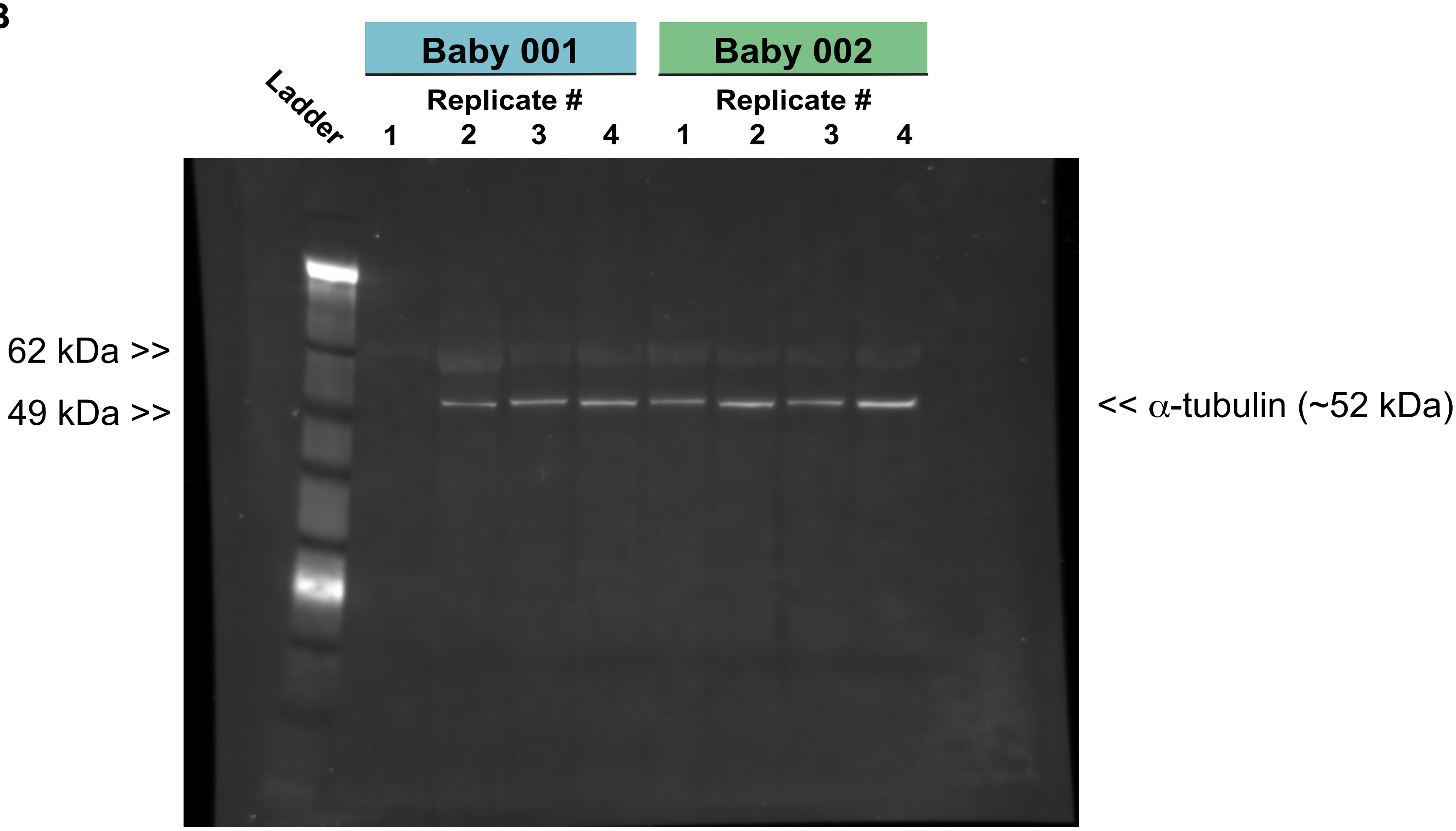

Supplemental Figure 7.

A

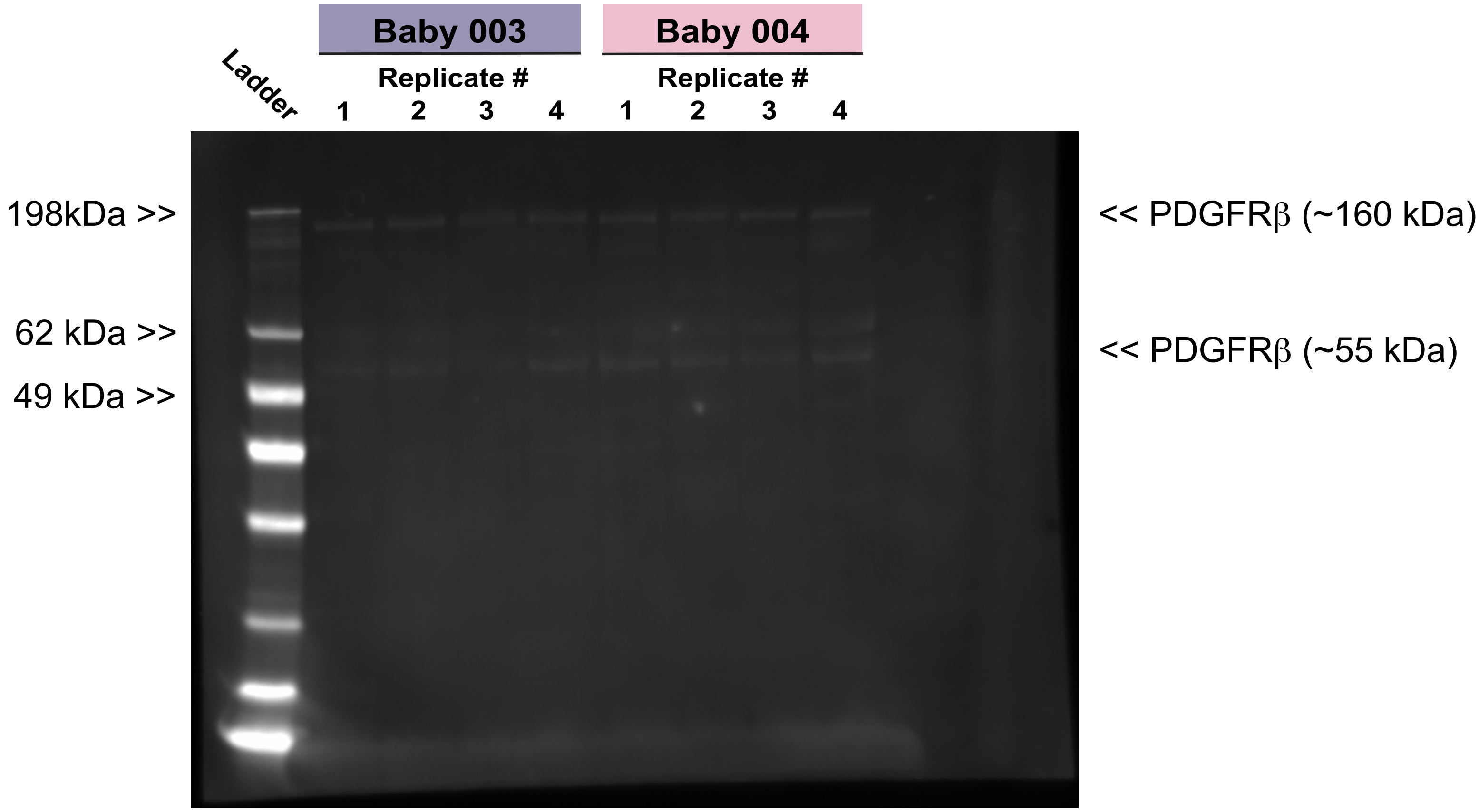

B

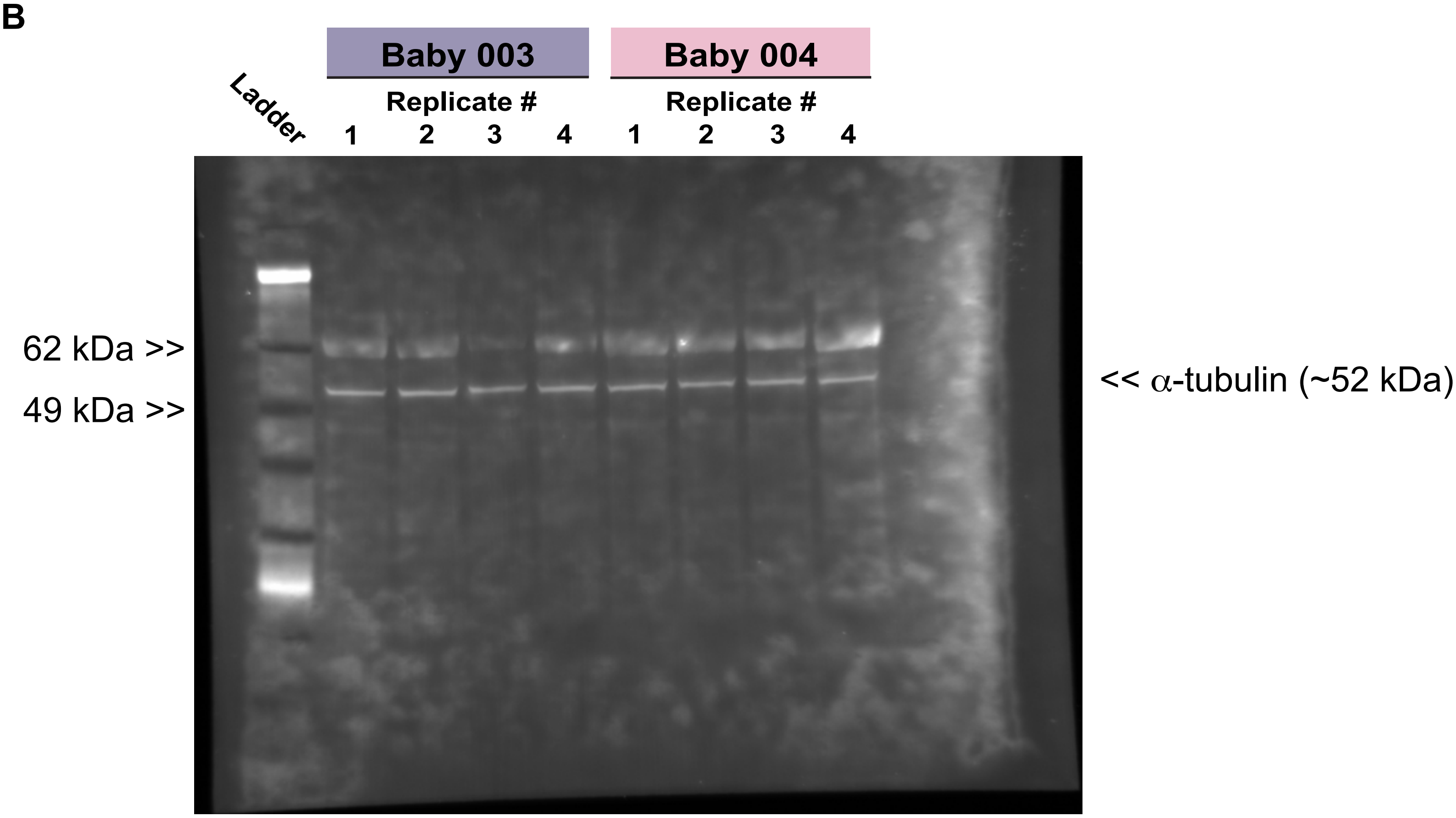

Supplemental Figure 8.

A

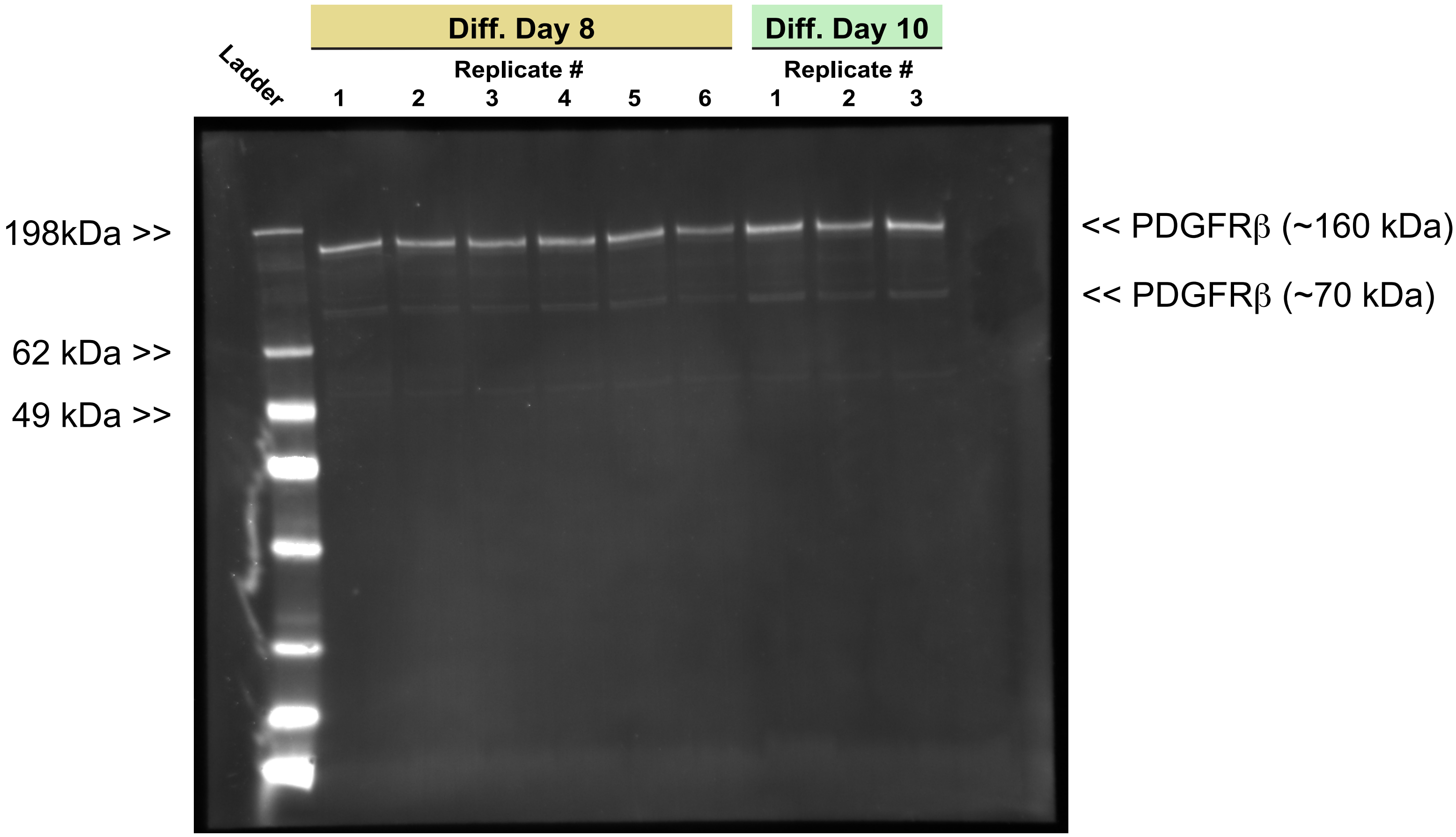

B

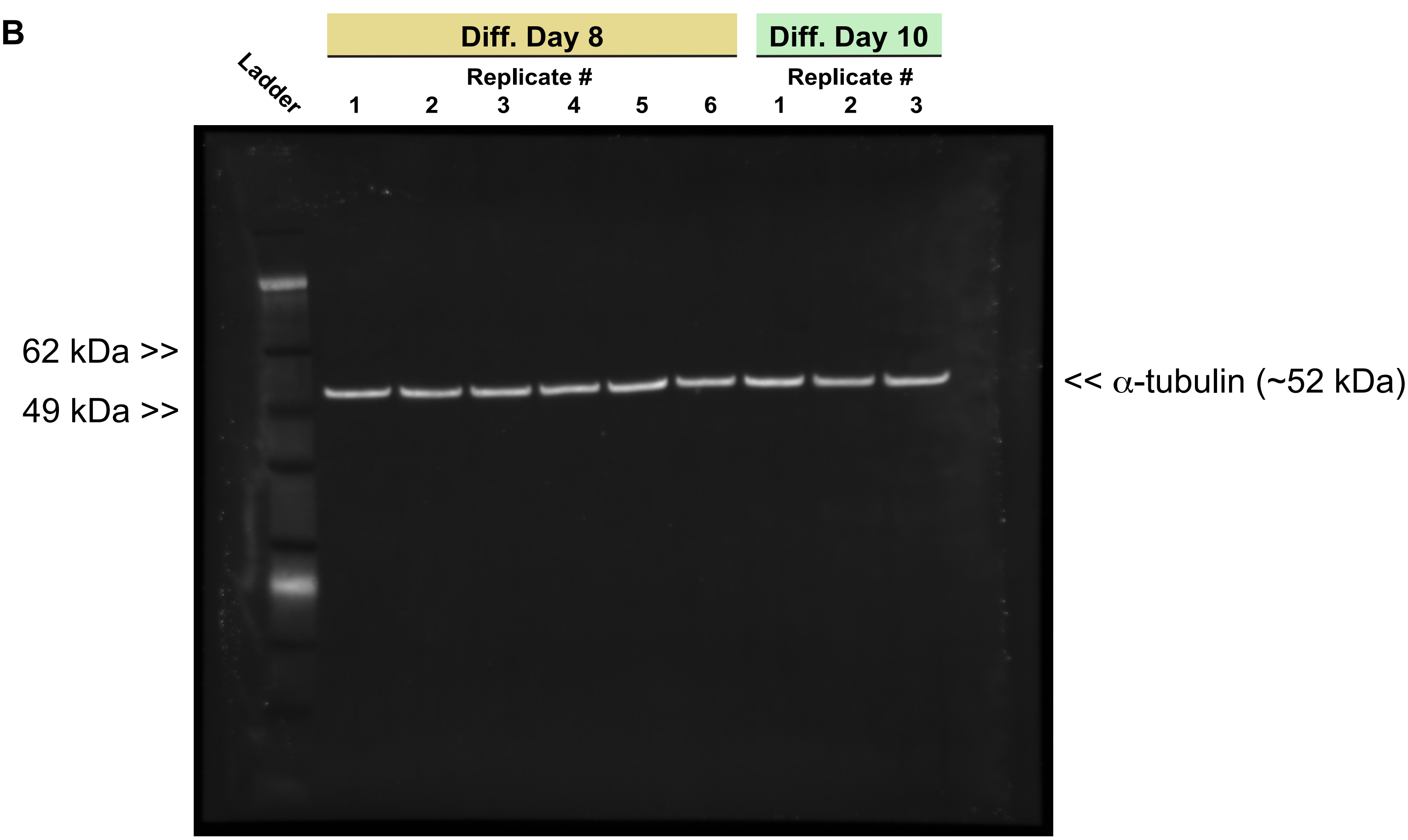

Supplemental Figure 9.

A

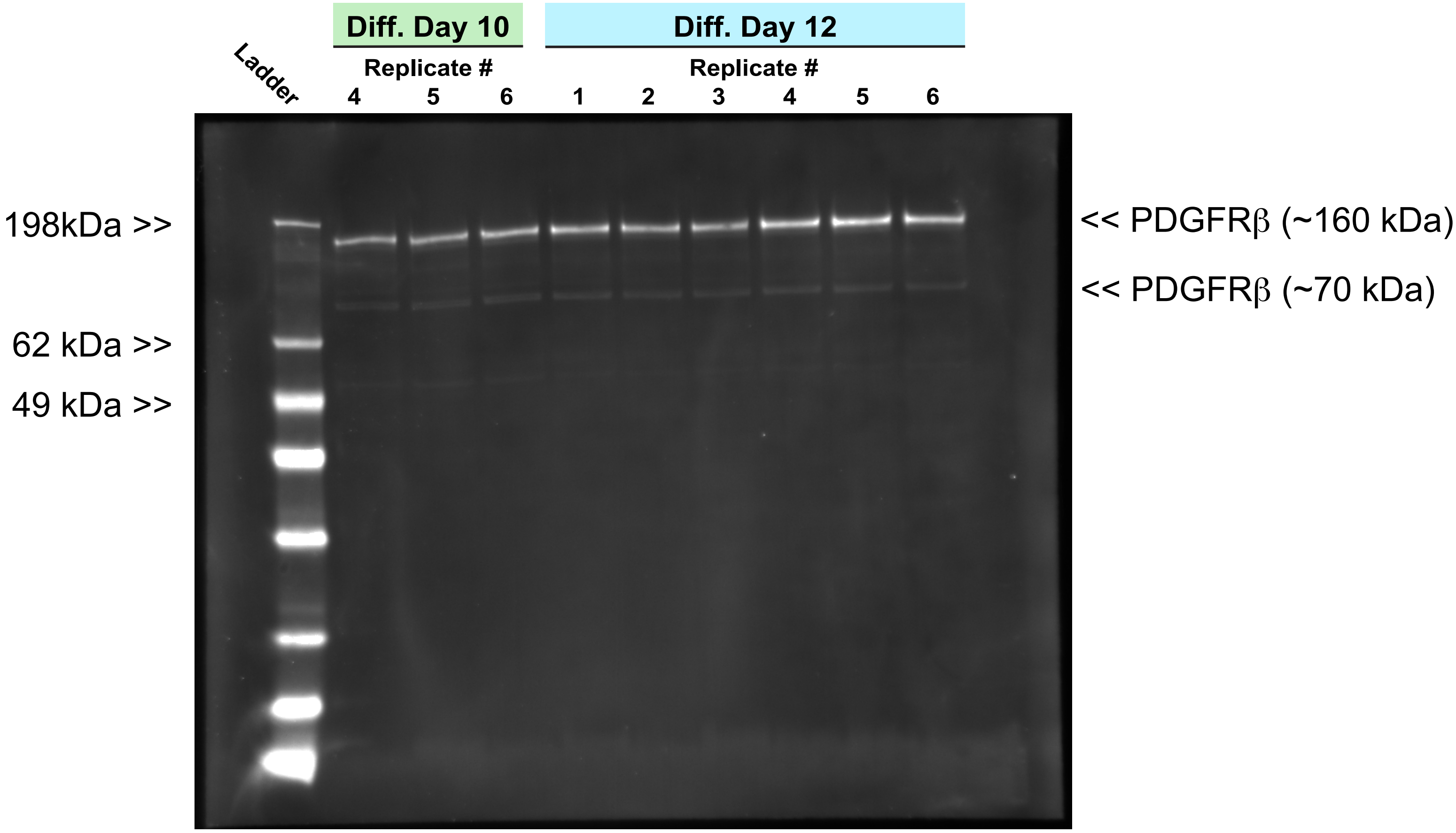

B

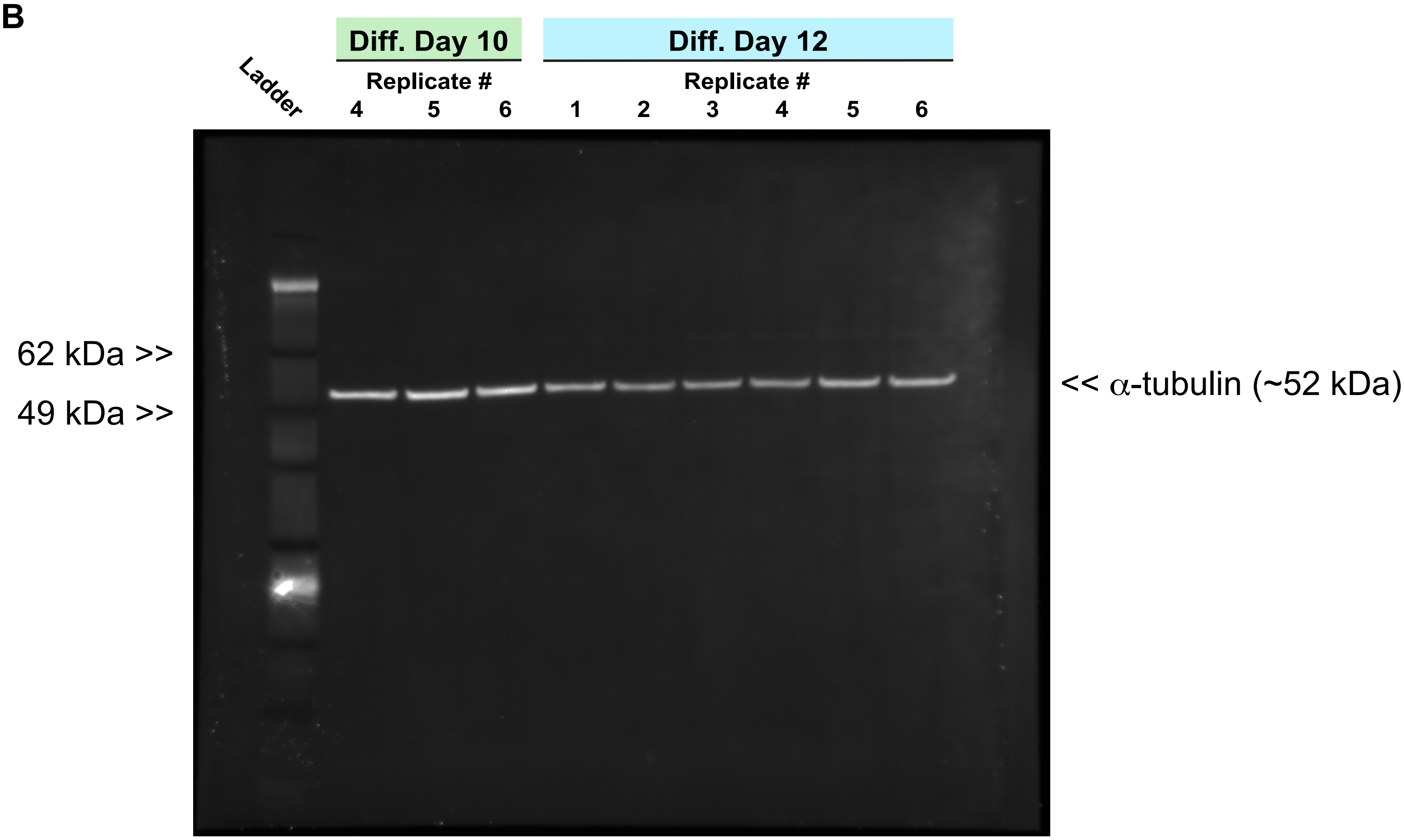

Supplemental Figure 10.

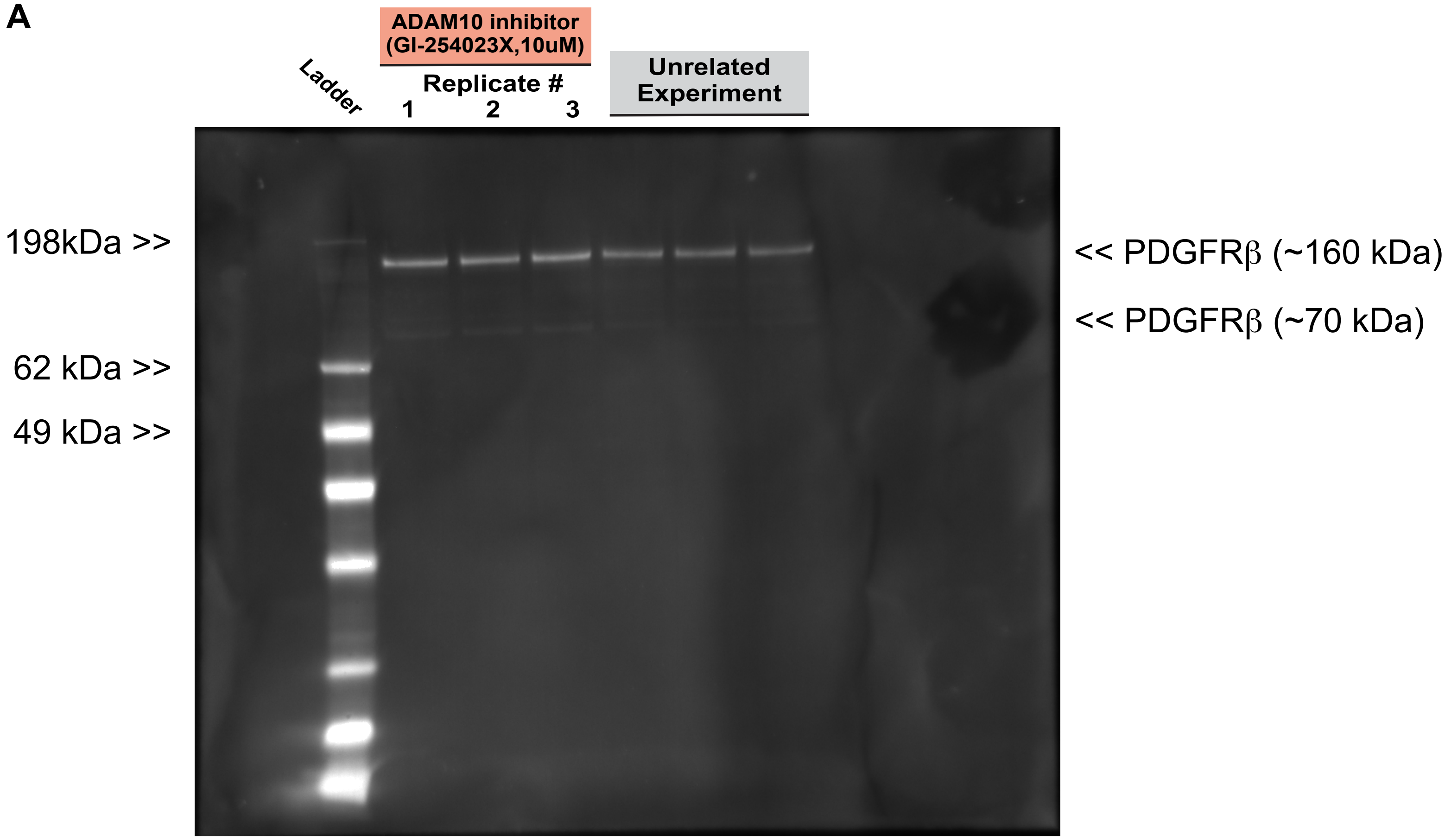

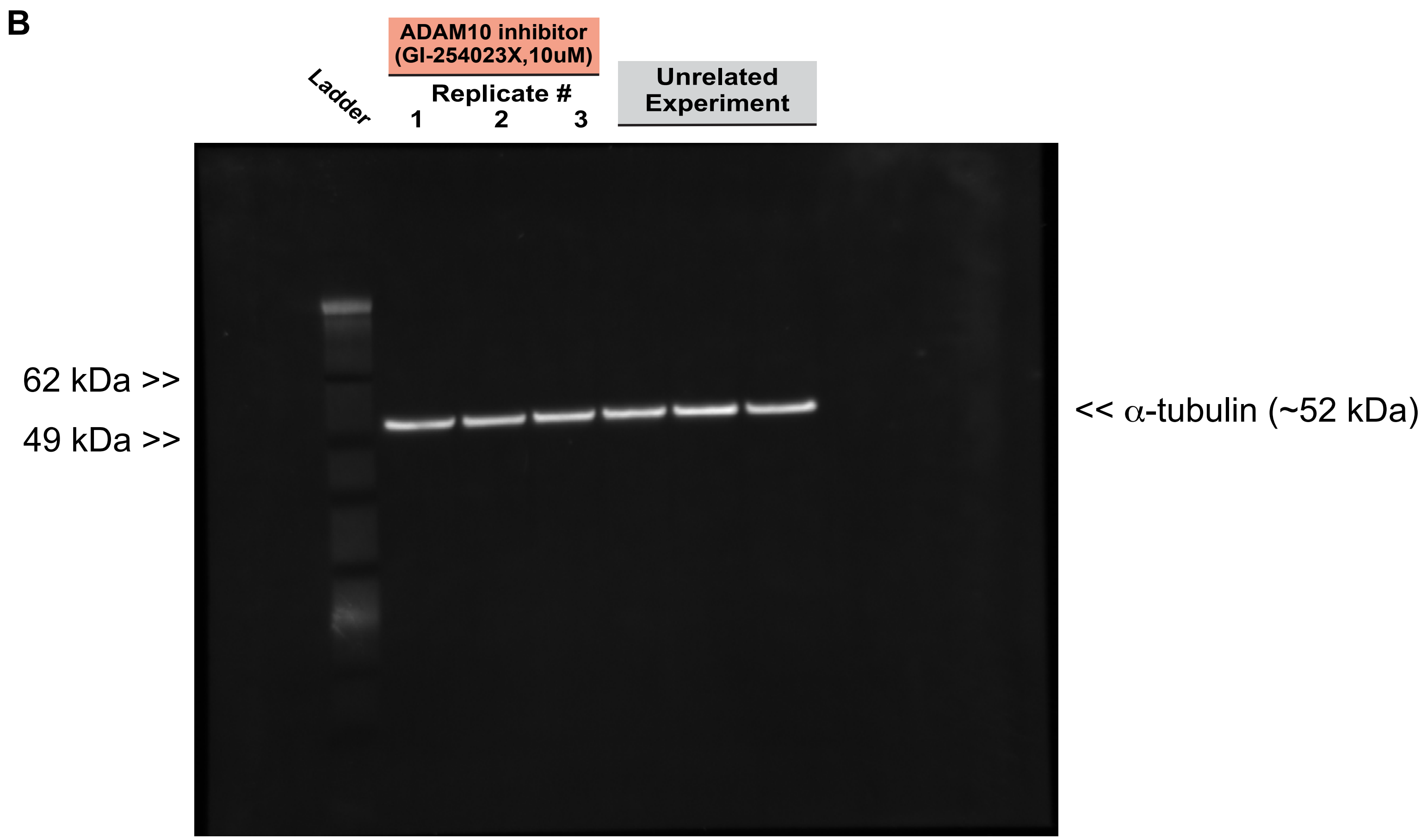

Supplemental Figure 11.

A

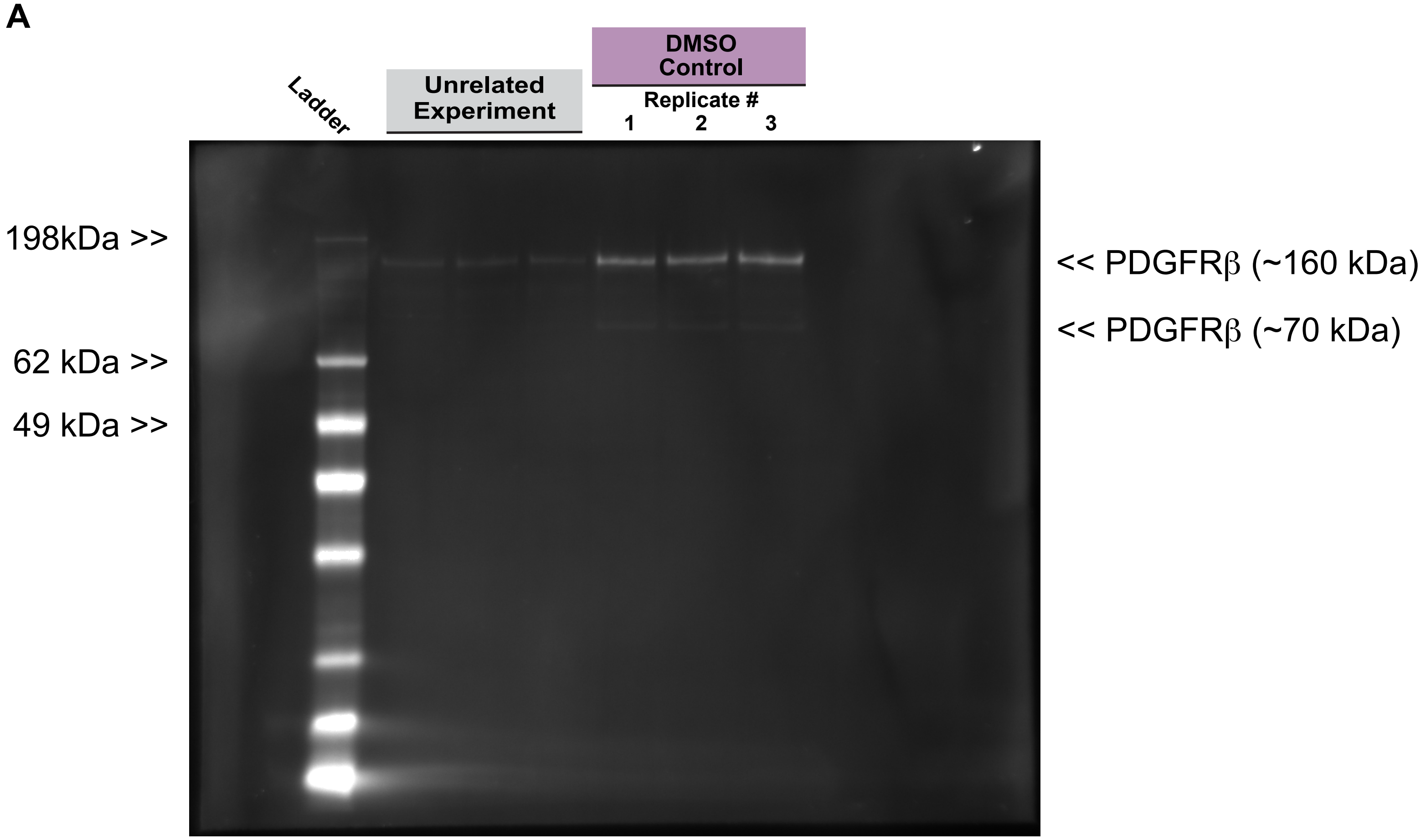

B

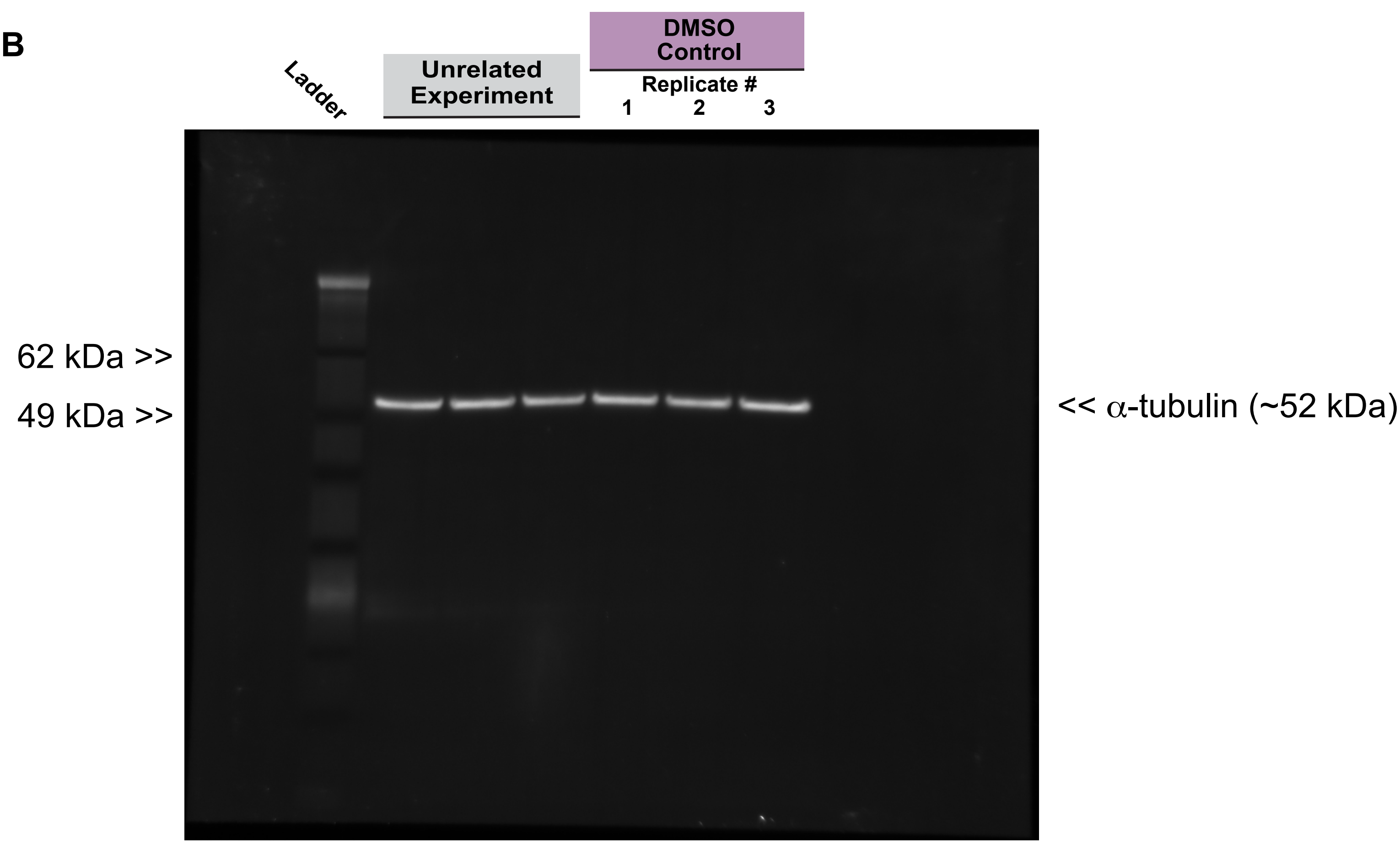

Supplemental Figure 12.

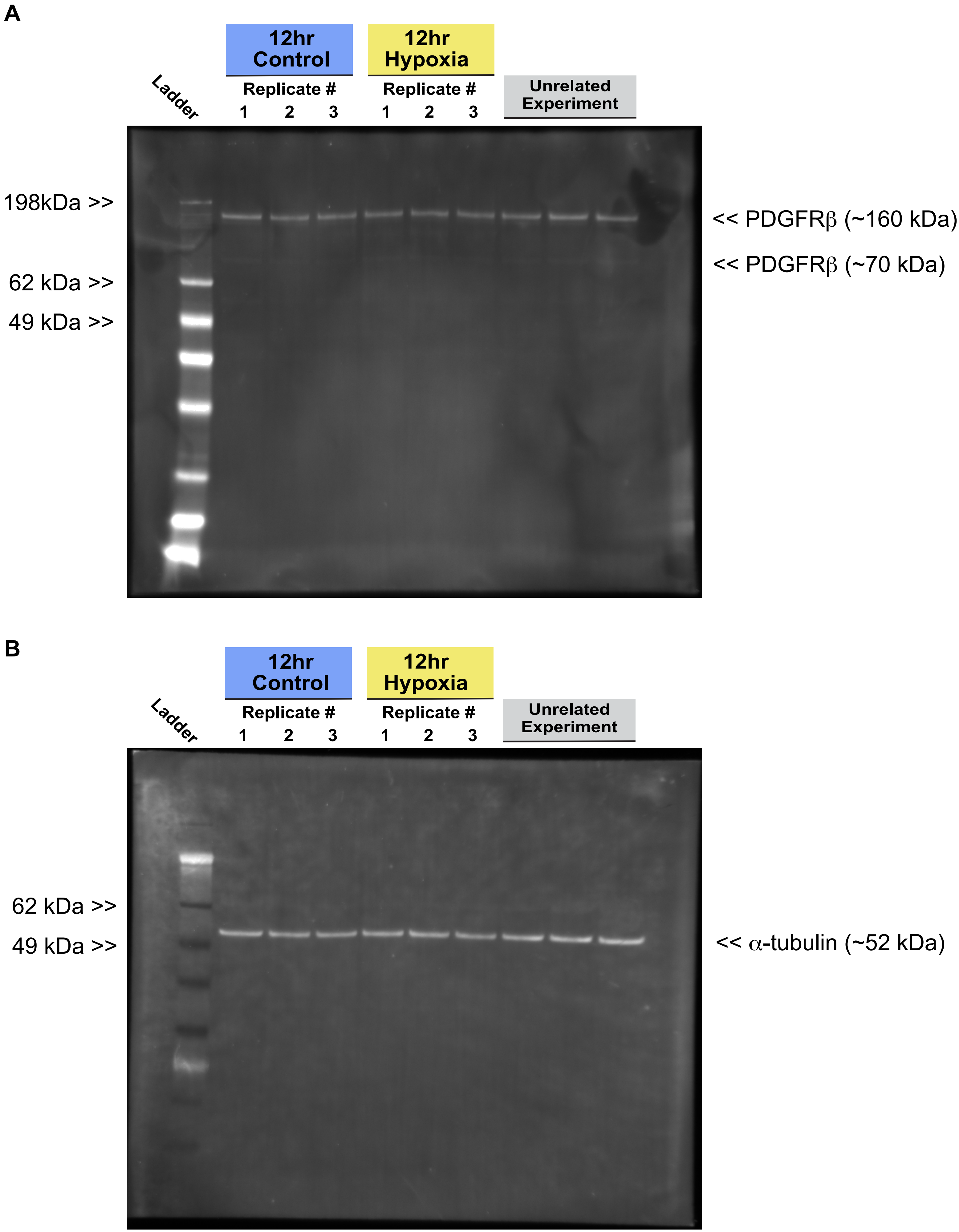

Supplemental Figure 13.

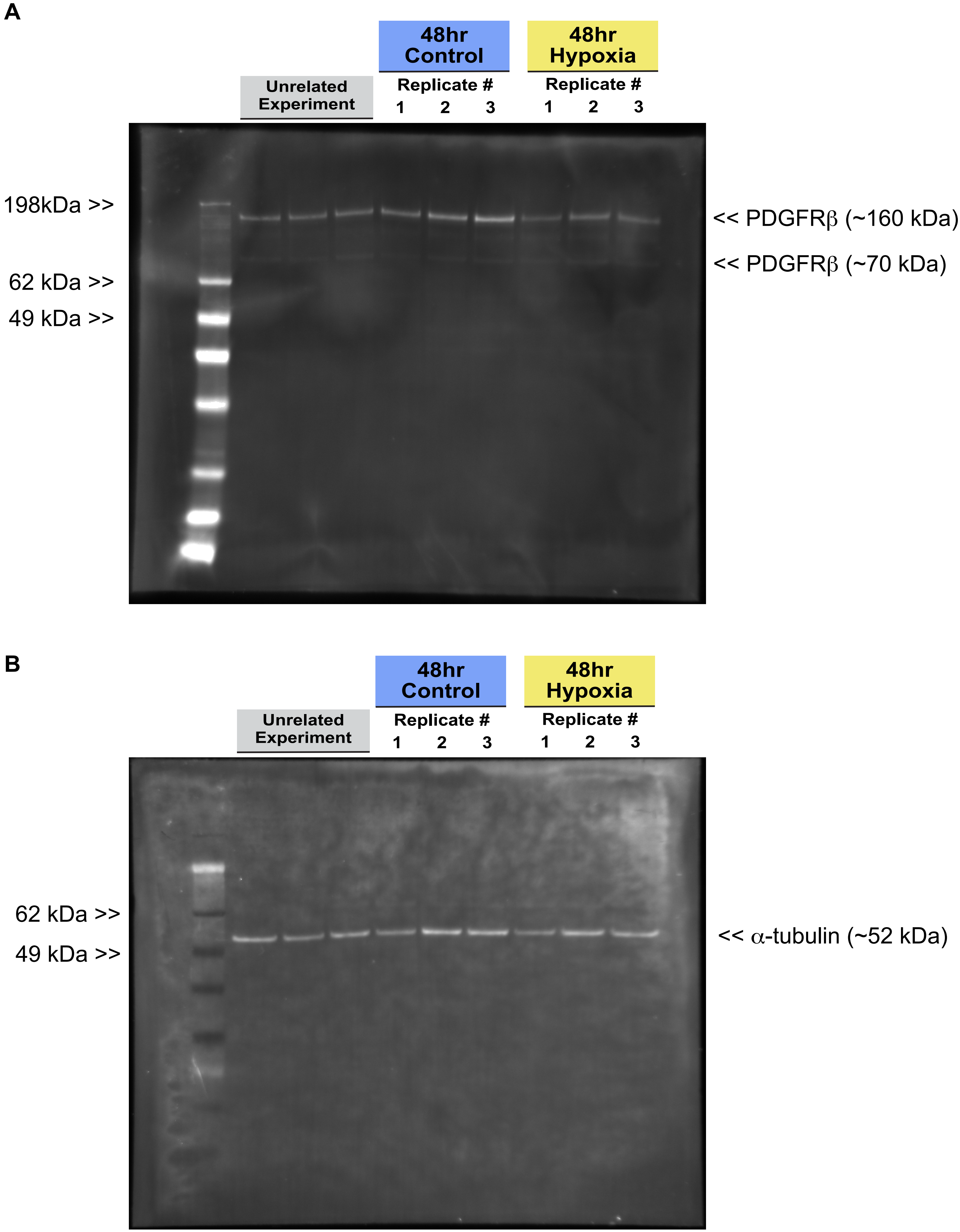

Supplemental Figure 14.

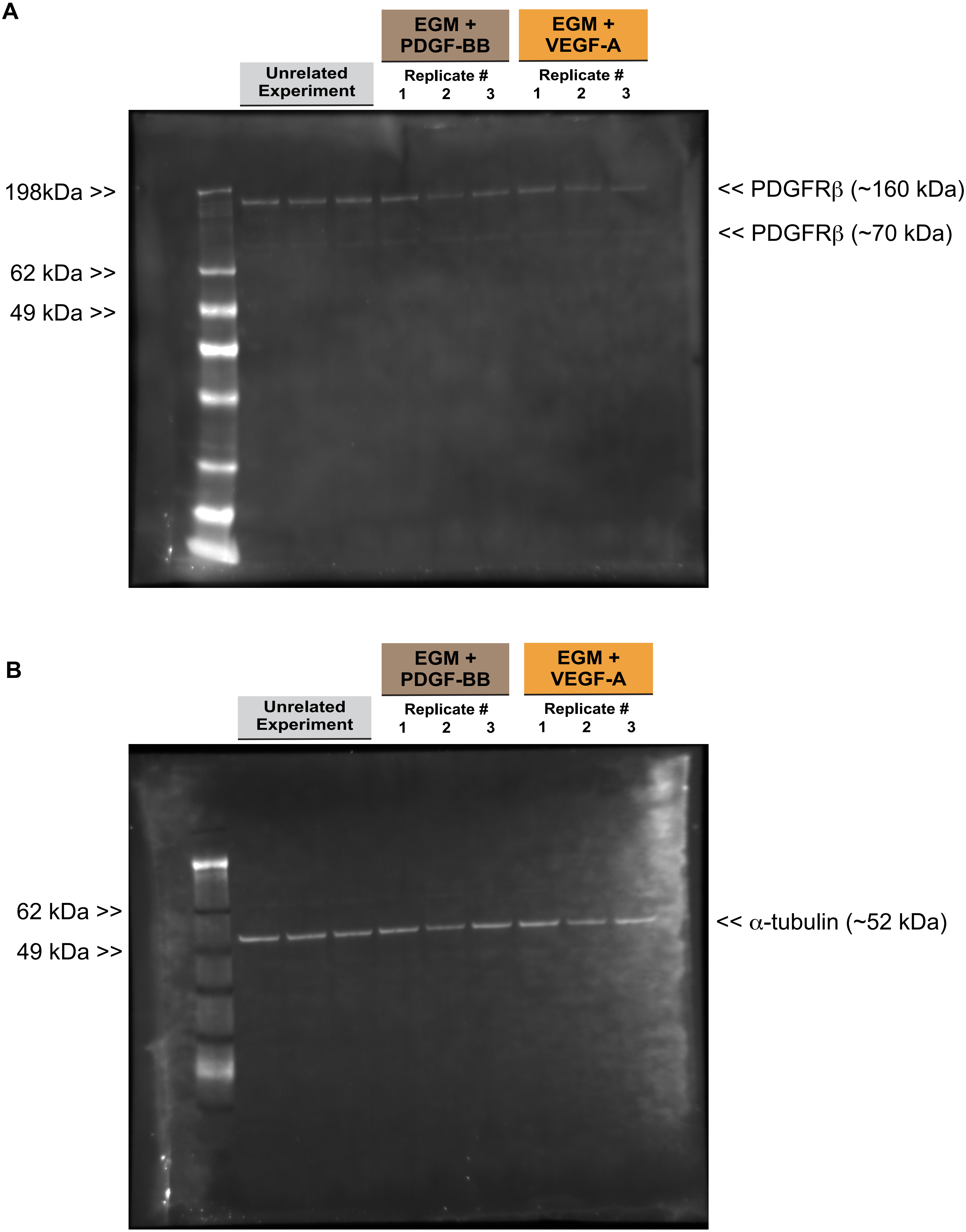

Supplemental Figure 15.

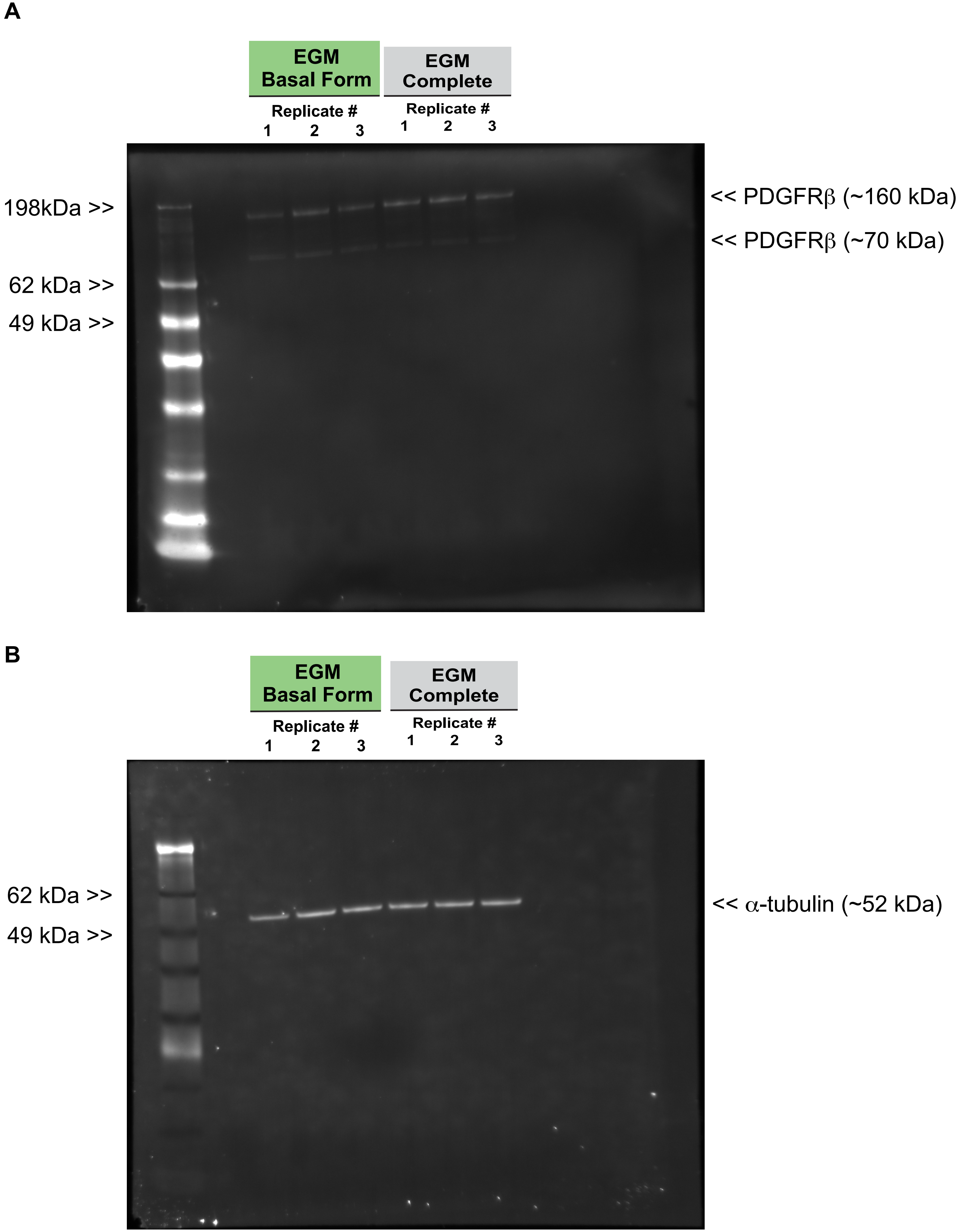

Supplemental Figure 16.

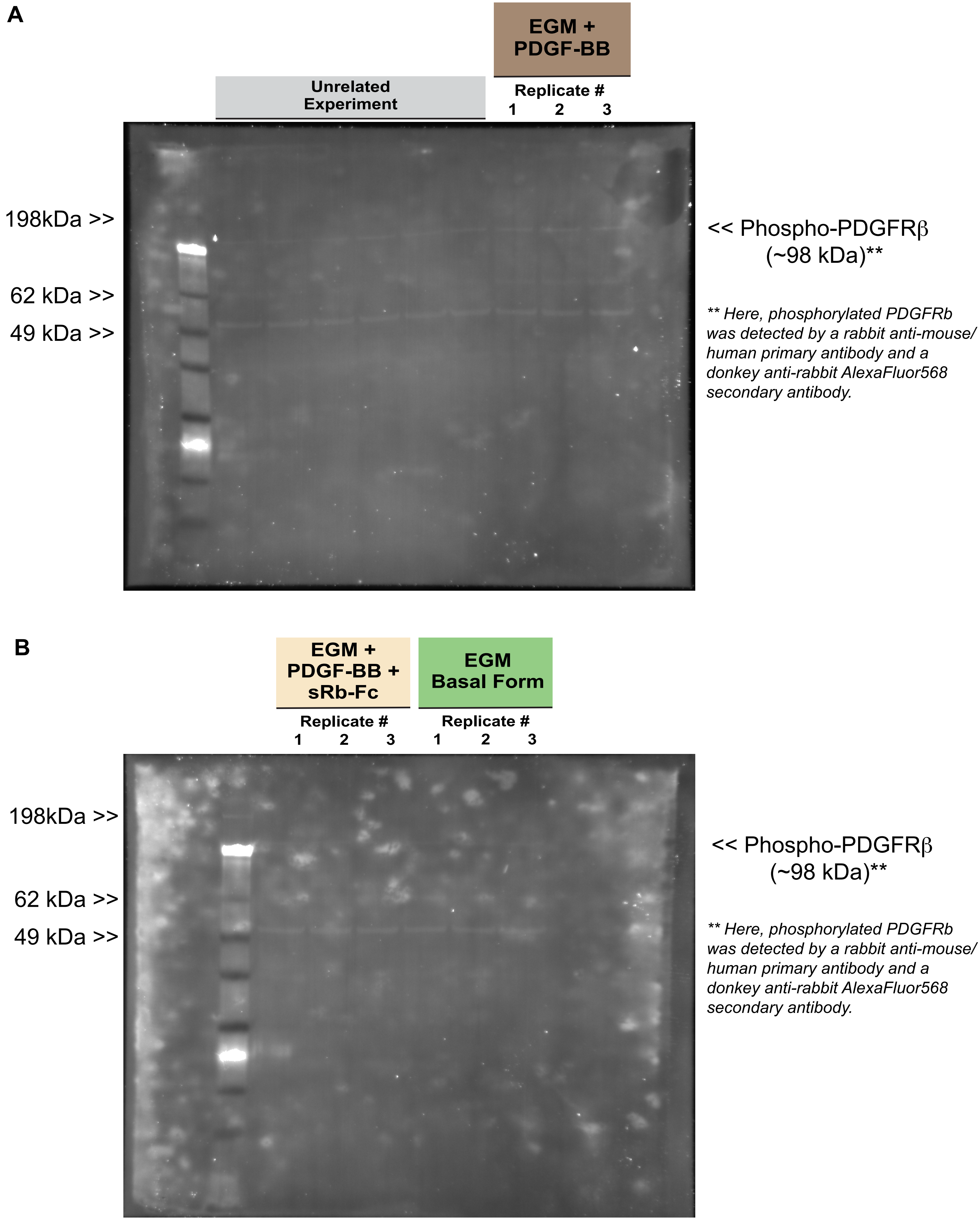

Supplemental Figure 17.

A

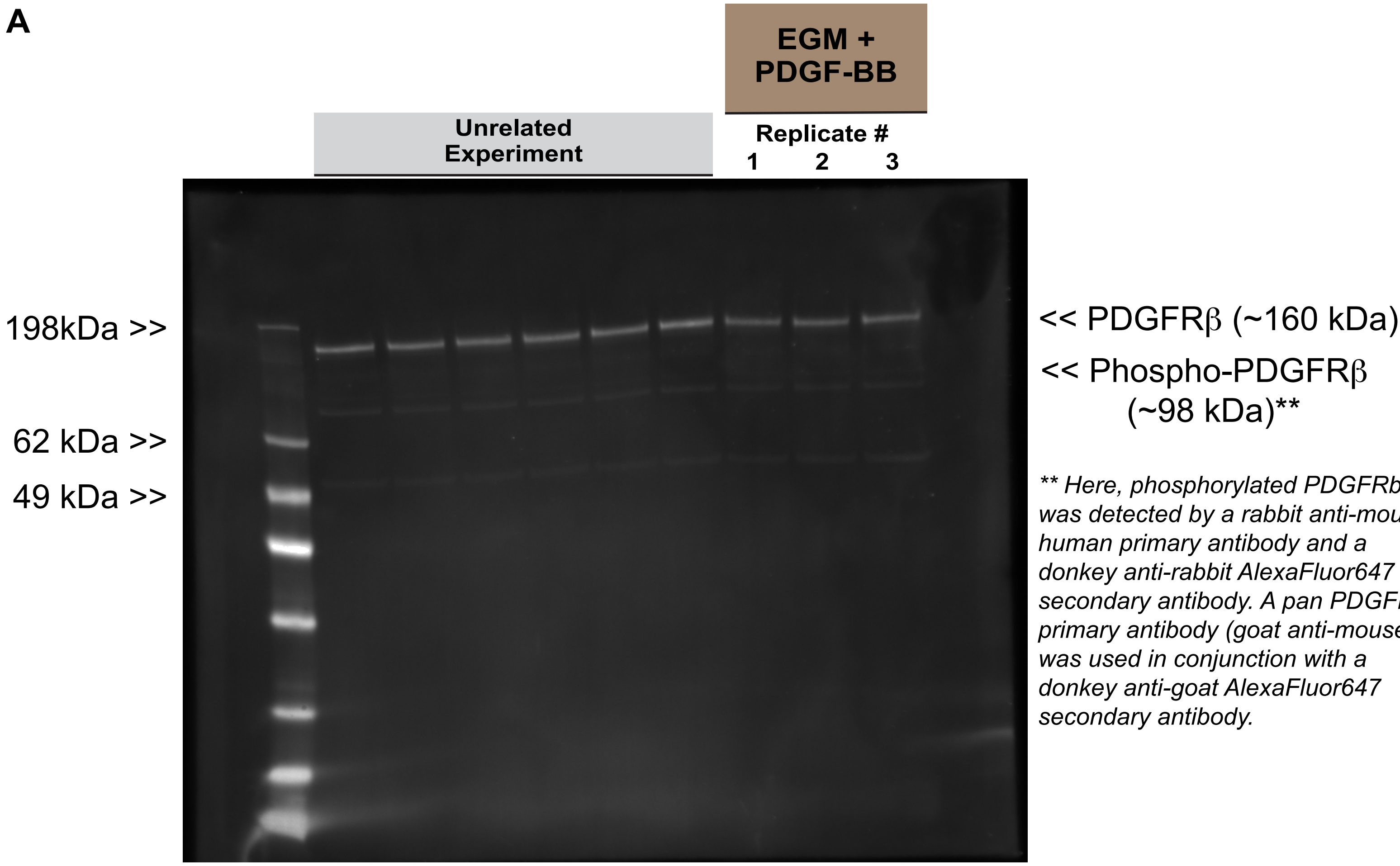

*** Here, phosphorylated PDGFRb was detected by a rabbit anti-mouse/ human primary antibody and a donkey anti-rabbit AlexaFluor647 secondary antibody. A pan PDGFRb primary antibody (goat anti-mouse) was used in conjunction with a donkey anti-goat AlexaFluor647 secondary antibody.*

B

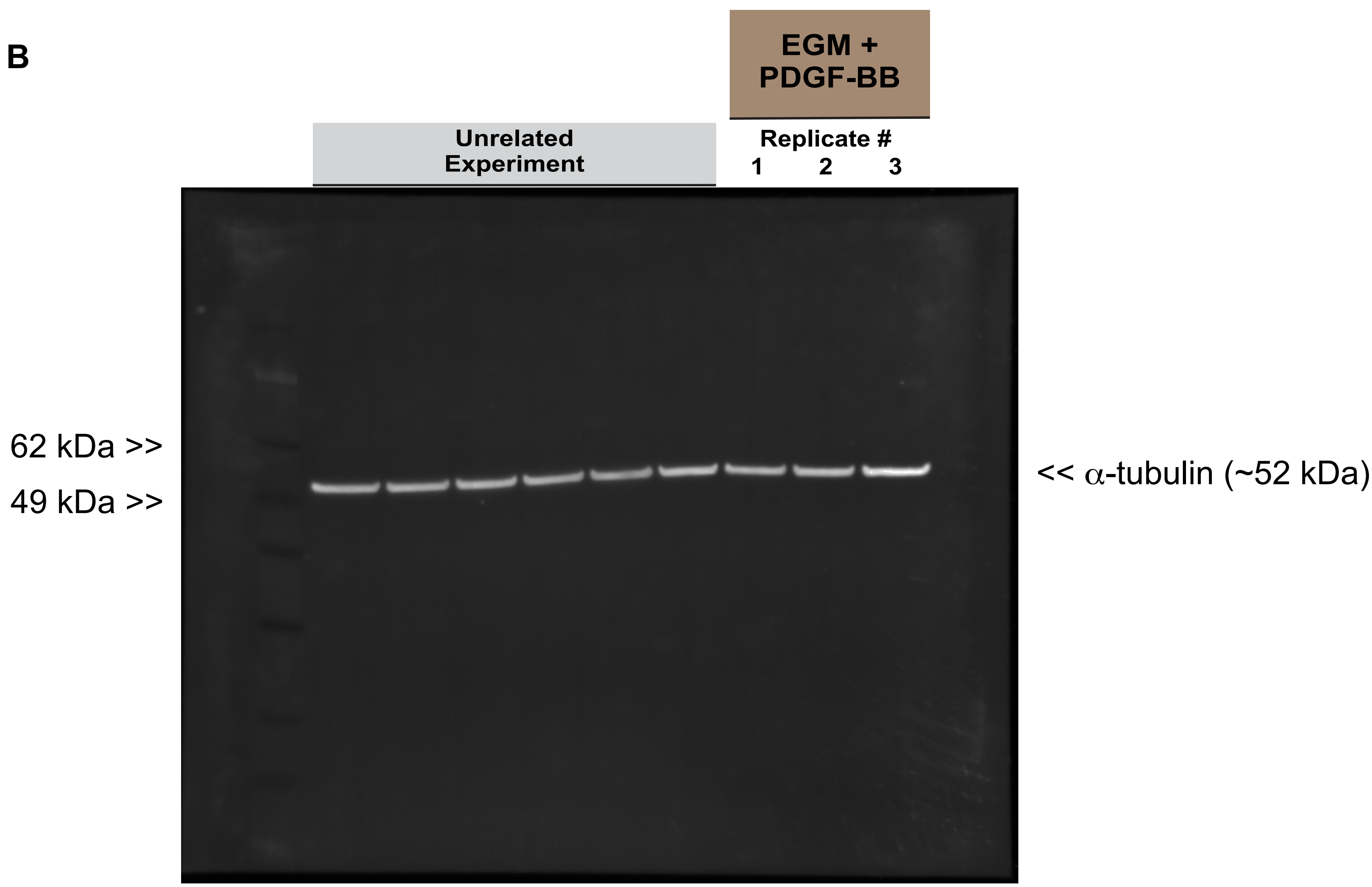

Supplemental Figure 18.

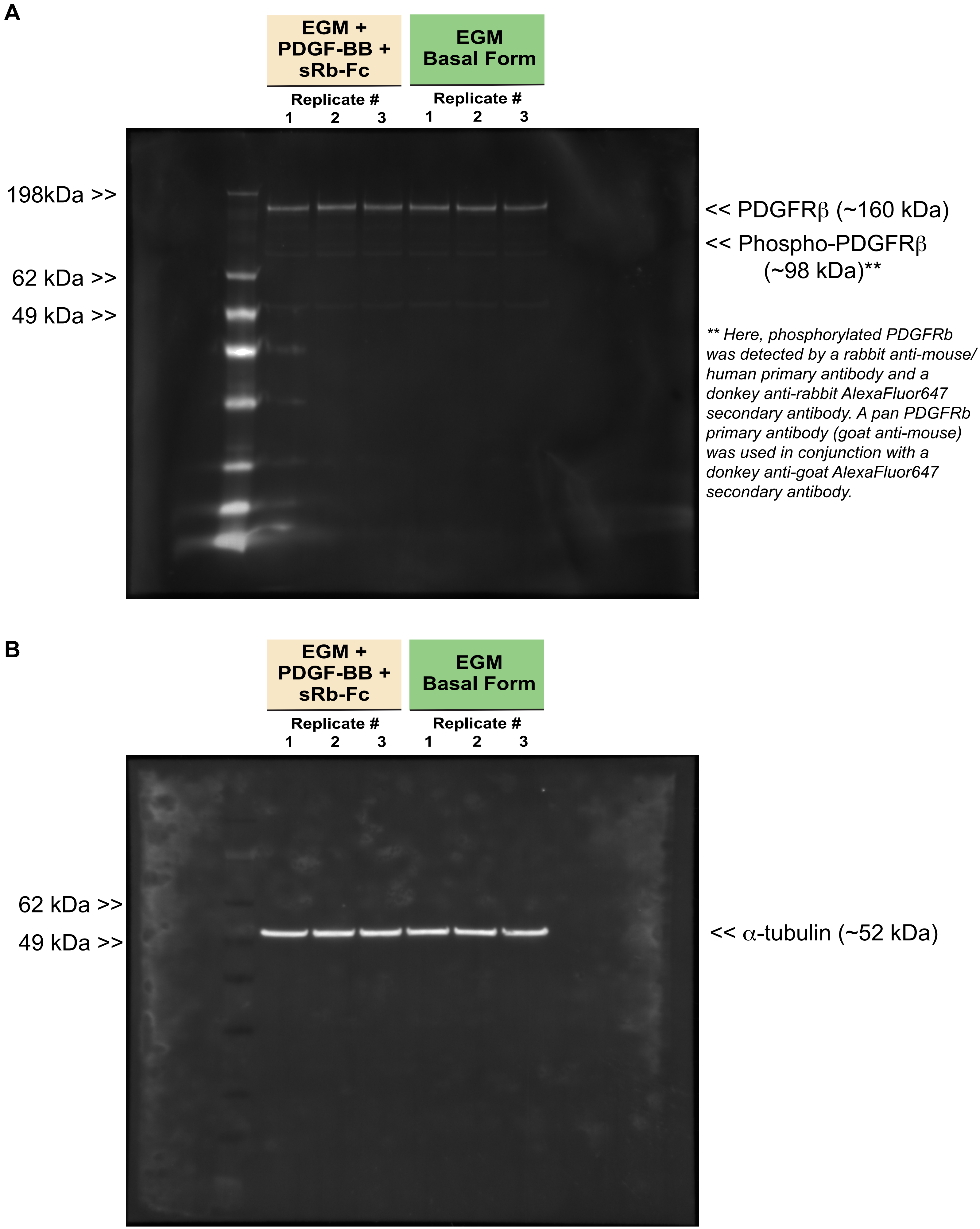

Supplemental Figure 19.
